# Natural Clearance of Prolonged VDPV Infection in a Child With Primary Immunodeficiency Disorder

**DOI:** 10.3389/fimmu.2019.01567

**Published:** 2019-07-23

**Authors:** Madhu Chhanda Mohanty, Manisha Ranjan Madkaikar, Mukesh Desai, Jahnavi Aluri, Swapnil Yashwant Varose, Prasad Taur, Deepa Kailash Sharma, Uma Prajwal Nalavade, Sneha Vijay Rane, Maya Gupta, Snehal Shabarish, Aparna Dalvi, Jagadish Mohanrao Deshpande

**Affiliations:** ^1^ICMR-National Institute of Virology, Mumbai Unit, Formerly Enterovirus Research Centre, Indian Council of Medical Research, Mumbai, India; ^2^ICMR-National Institute of Immunohaematology, Indian Council of Medical Research, KEM Hospital, Mumbai, India; ^3^Bai Jer Bai Wadia Childrens Hospital, Mumbai, India

**Keywords:** primary immunodeficiency disorder, oral polio vaccine, vaccine-derived polioviruses, severe combined immune-deficiency, leaky SCID, inflammatory cytokines

## Abstract

The emergence of immunodeficiency-associated vaccine-derived polioviruses (iVDPV) from children with primary immunodeficiency disorders poses a threat to the eradication program. Herein, we report a patient with severe combined immunodeficiency (SCID), identified as a prolonged serotype 3 iVDPV (iVDPV3) excreter with 13 VDPV3 isolates and a maximum of 10.33% nucleotide divergence, who abruptly cleared infection after a period of 2 years. Occurrence of an episode of norovirus diarrhea associated with increased activated oligoclonal cytotoxic T cells, inverse CD4:CD8 ratio, significantly elevated pro-inflammatory cytokines, and subsequent clearance of the poliovirus suggests a possible link between inflammatory diarrheal illness and clearance of iVDPV. Our findings suggest that in the absence of B cells and sufficiently activated T/NK cells, macrophages and other T cells may produce auto-inflammatory conditions by TLR/RLR ligands expressed by previous/ongoing bacterial or viral infections to clear VDPV infection. The study highlights the need to screen all the patients with combined immunodeficiency for poliovirus excretion and intermittent follow-up of their immune parameters if found positive, in order to manage the risk of iVDPV excretion in the polio eradication endgame strategy.

## Introduction

Oral poliovirus vaccine (OPV) usage has decreased the annual incidence of wild-type poliomyelitis >99.9% worldwide (1, 2). Despite the effectiveness and benefits of OPV administration to eradicate wild polioviruses, OPV use comes with some risks (1, 3). In polio-free countries, there is a risk of importation of wild-type poliovirus and vaccine-derived poliovirus (VDPV) that has mutated from OPV-associated strains and potentially regained neurovirulence (4, 5). By definition, VDPV1 and VDPV3 show >1% and VDPV2 shows >0.6% nucleotide divergence in viral protein 1 (VP1) coding region, as compared with their parental OPV strains (6). Patients with primary immunodeficiency (PID) are at ~3,000-fold higher risks of developing VDPV infection (7, 8). These patients may excrete iVDPVs for a long time (reported up to >28 years), even in the absence of any clinical symptom (9, 10). Prolonged shedding of neurovirulent iVDPVs can potentially cause poliomyelitis outbreaks when the level of population immunity is reduced (5, 11, 12).

As part of our study on poliovirus infection in immunodeficient children in India, we had identified and reported a severe combined immunodeficient (SCID) patient with asymptomatic and prolonged iVDPV infection who abruptly stopped poliovirus shedding after 2 years of continuous excretion (13). Here, we report the virological and molecular characterization of the sequential isolates and the immunological parameters of the patient in relation to poliovirus clearance.

### Case Presentation

The patient is a male child born in Mumbai in July 2010 with no significant illness in the history of the family and no siblings. He had received the routine (at birth) as well as the pulse polio doses up to 14 months of age (Table 1A). Other than the birth doses, the actual doses of OPV received (number, time, and serotype) are not known. A clinical scenario of respiratory, gastrointestinal, and urinary tract infections with severe failure to thrive prompted the evaluation of an underlying immunodeficiency in the child. He was referred to the National Institute of Immunohematology (NIIH) at the age of 14 months to rule out PID. A finding of T low, B low, and normal NK cell phenotype led to a suspicion of SCID. SCID represents one of the most severe forms of PID and is associated with severe defect in the T cell compartment with an effect on the B and NK cell number and/or function. According to the Primary Immunodeficiency Treatment Consortium (PIDTC), SCID is further categorized into Typical SCID, Leaky SCID, and Omenn Phenotype. In view of T cell counts > 300 cells/μl, the child was categorized into Leaky SCID at the age of 16 months and was evaluated for thymic output (14). He had low naïve T cell population, reduced T cell receptor excision circle, and hypogammaglobulinemia, which further aided the diagnosis of SCID and was eventually screened for a genetic defect in the RAG1/RAG2 gene. However, no mutation was identified in these genes. T cell functionality was tested and found to be abnormal. A clinical exome panel was performed; however, no mutation was identified in any of the SCID/CID genes.

**Table 1A:** Characteristics of the SCID patient excreting vaccine-derived poliovirus.

**Characteristics**	**VDPV patient**
Age at hospitalization, months	48
Sex	Male
Immunodeficiency type	Severe combined immunodeficiency (SCID)
Diagnosis of immunodeficiency (months)	16
OPV doses	Routine (birth, 6, 10, and 14 weeks) pulse polio up to 14 months of age
IPV^*^ dose (age at vaccination in months)	42
Other vaccinations	DPT^†^, hepatitis, and measles
Period of poliovirus excretion after 1st detection, months	24
Estimated total time of virus excretion at the time of detection, months	~48
Estimated total time of poliovirus excretion, months	73
Maximum nucleotide differences^‡^	93
Neutralizing antibody tires^§^	
Against poliovirus type 1	6
Against poliovirus type 2	7
Against poliovirus type 3	28

* *Inactivated polio vaccine*.

† *Diphtheria, pertussis tetanus (vaccine)*.

‡ *With parent Sabin strain*.

§ *Reciprocate titers 1:6, 1:7, 1:28*.

Serological investigations confirmed below normal levels of immunoglobulins. He has been receiving intravenous immunoglobulin therapy (IVIG) since the age of 16 months.

The child experienced several episodes of dysentery, loose motion, and respiratory distress, for which he was admitted to the Wadia Hospital several times (Table 1B). A total of 27 fecal specimens were obtained for virological examination between September 2014 and July 2018 (from 4 to 8 years of age) at approximately monthly intervals, from which the first 13 specimens were found positive for poliovirus. After a prolonged excretion for 2 years, the child abruptly stopped poliovirus excretion; subsequent collections were found negative for poliovirus. He has not received OPV after the diagnosis of SCID; however, he has been immunized with IPV at the age of 3.5 years. Bone marrow transplant (BMT) has not been performed to date.

**Table 1B:** Clinical presentations of the SCID patient during hospital admissions.

**Age in months**	**Clinical diagnosis**
11	Bacillary dysentery
14	Tuberculosis
24	Pneumonia
30	Loose motion (*Cryptosporidium*)
48	UTI^*^
54	UTI
60	Pneumonia, UTI (*Enterobacter*)
70	Hypocalcaemia
73	Persistent loose motion, diarrhea (Norovirus), CMV^†^, anemia
80	Acute gastroenteritis with anemia
92	Loose motion, high-grade fever

* *Urinary tract infection*.

† *Cytomegalovirus positive*.

## Materials and Methods

### Immunological Workup

The study has been conducted in collaboration with the clinicians of PID surveillance group at NIIH, Mumbai and Bai Jerbai Wadia Children's Hospital (BJWH), Mumbai, India. Ethical clearance was obtained from the Ethics Committee of NIIH, BJWH, and NIVMU (National Institute of Virology, Mumbai Unit). Blood samples were collected at BJWH as part of routine surveillance; diagnosis of PID was performed at NIIH.

The investigations were guided by the clinical presentation, immunological abnormalities, and molecular diagnosis according to the phenotypic classification of the International Union of Immunological Societies (IUIS) (13, 15).

Lymphocyte subset analysis was performed by flow cytometry by using BD Multitest 6-color TBNK reagent followed by acquisition of cells on FACS Aria I using FACS Diva Software (BD Biosciences, San Jose, CA, USA). The patient was evaluated for cell surface markers specific for T cells (anti-CD3 Peridinin-chlorophyll-protein Complex: CY5.5 Conjugate; PerCP-Cy5), B cells (anti-CD19 allophycocyanin; APC), monocytes (anti-CD14), and anti HLA-DR (anti-HLA DR fluorescein isothiocyanate; FITC). The percentages of naive T cell subsets on CD4 and CD8 cells were measured by flow cytometric evaluation of CD45RA, CD62L using anti-CD45RA phycoerythrin (PE), and anti-CD62L allophycocyanin (APC) purchased from BD Biosciences, San Jose, CA, USA. To maximize instrument performance and minimize inter-assay variability, CST (Cytometer Set-up and Tracking, BD, USA) beads were run daily on a BD Facs Aria machine. Oligoclonality of T cell repertoire was assessed for research purposes by using the IOTest® Beta Mark. T cell receptor excision circles (TRECs) were measured using an in-house modification of the previously described method (16). The information regarding HLA-DR expression, T-memory cell workup, and TREC assay is provided in Supplementary Appendix.

### Estimation of Serum Immunoglobulin Levels

Serum immunoglobulin concentration (IgG, A, M, and E) was estimated by nephelometry (BN Prospec, Siemens, Germany) as per the manufacturer's instruction. Quantification of serum IgG and IgA by ELISA was performed by using rabbit polyclonal anti-human IgG (A-0423, Dakopatts, Denmark), peroxidase-conjugated rabbit anti-human IgG (P-0214), rabbit polyclonal anti-human IgA (A-0262 Dakopatts, Denmark), and peroxidase-conjugated rabbit anti-human IgA (P-0216, Dakopatts, Denmark) as coating and detecting antibodies for IgG and IgA sandwich ELISA as described earlier (17).

### Fecal Sample Collection, Processing, and Enterovirus Isolation

The fecal sample processing, culture, virus isolation, and characterization of virus-positive isolates were performed at NIVMU as described earlier as per WHO laboratory manual (13, 18). We used human rhabdomyosarcoma (RD) and transgenic mouse cell line expressing polio receptor (L20B) for enterovirus culture. Cells were infected with fecal extract and cytopathic effect (CPE) was observed for 5 days. The samples were scored as negative if two consecutive passages in the same cell line did not produce CPE.

Real-time PCR was used for intratypic differentiation of the isolates. All the isolates were further characterized using VP1 (900 nt) region sequencing.

### RT-PCR and Sequencing

Viral RNA was extracted from freeze–thaw lysate of infected RD cell culture supernatant using QIAamp Viral RNA Mini kit (QIAGEN, Chatsworth, CA). For VP1 region amplification (900 nt), reverse transcriptase PCR was carried out in a single tube using reverse primers Q8 and forward primer Y7R as described earlier (19). PCR amplicons of the desired lengths were excised from agarose gel following electrophoresis and purified using QIA quick Gel Extraction kit (QIAGEN, Chatsworth, CA). Big Dye Terminator v3.1 Cycle Sequencing Kit (Applied Bio Systems, Foster city, CA) was used for sequencing as per the manufacturer's instructions (http://www.appliedbiosystems.com). Sequences were resolved on an ABI 3130xl Genetic Analyzer (Applied Biosystems, Foster City, CA) and edited using Sequencher v4.10.1 software (Gene Codes, USA).

### Comparative Analysis of Nucleotide and Amino Acid Sequences

The VP1 sequences of poliovirus reference strains were obtained from the GenBank database. Alignment of VP1 region was performed by the program CLUSTAL W (http://www.ebi.ac.uk) embedded in MEGA7 (http://www.megasoftware.net). Pairwise nucleotide and amino acid sequences were analyzed and compared.

### Poliovirus Neutralizing Antibody Titer

Poliovirus neutralizing antibody levels of the patient were determined using the standard micro-neutralization assay (20). Sabin poliovirus vaccine strains obtained from NIBSC, UK, were used as challenge virus and HEp-2 Cincinnati cells were used as the cell substrate. Serial 2-fold dilutions (1:8–1:1,024) of the serum samples were used.

### Multiplex Cytokine Assay

Multiplex cytokine analysis kits (Merck, Milliplex) were used for cytokine assays and run in duplicate according to the manufacturer's protocol. Fourteen cytokines/chemokines, including pro-inflammatory (IL-1β, IL-6, IL-8, and TNF-α), Th1 (IFN-α, IFNγ, and TNFα), Th2 (IL-10, IL-4, and IL-13), and associated cytokines and chemokines (MCP-1, MIP-1α, IP-10, and RANTES), were analyzed in serum samples using the Luminex-100 system Version 1.7 (Luminex, Austin, TX). Data analysis was performed using the MasterPlex QT 1.0 system (MiraiBio, Alameda, CA). A five-parameter regression formula was used to calculate the sample concentrations from the standard curves.

### Statistical Analysis

Student's *t*-test was used for comparing the mean cytokine values at different time points. *p* < 0.05 was considered significant. Sigma Plot was used for statistical analysis.

## Results

### Clinical Observations During the Period of Viral Clearance

During the 3-month period (73rd−75th month) in which the child cleared poliovirus infection, he was admitted to Wadia Hospital for 1 month (73rd month) for persistent diarrhea and anemia. The routine stool culture was bacteria negative, treated with the bactericidal drug meropenem for 21 days (Table 1B). The stored samples of this month were later on detected positive for cytomegalovirus (5,000 copies) and norovirus. He remained asymptomatic for the next 5 months. Previously, at the 70th month, he was admitted for hypocalcemia and started with vitamin D3 and calcium tablets.

### Neutralizing Antibody Titer

The serum samples collected at the age of 59 months were found to be positive for all three serotypes of poliovirus antibody but with a very low titer (Table 1A).

### Molecular Characteristics of iVDPV Isolates

**Figure 1:**
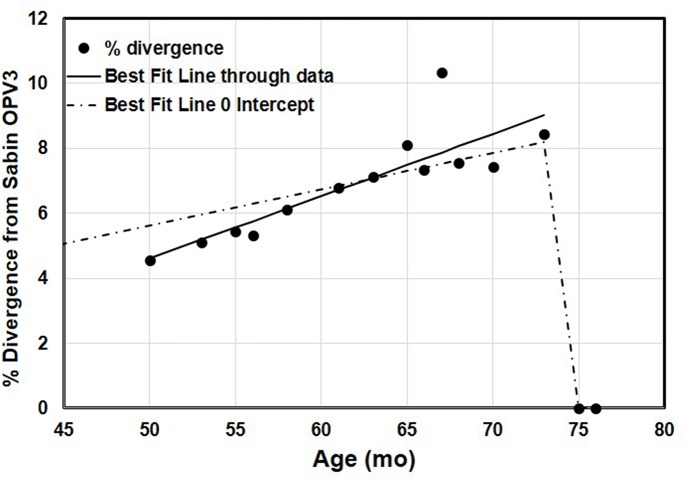
The accumulation of changes in the VP1 genomic region of 13 sequential poliovirus type 3 isolates under study. The actual observed data and the variability of the repeated measurements over the time of observation have been indicated. The data were adjusted to linear functions for the accumulation of substitutions. The two lines show the best-fitting linear trend to the observed positive data with and without an intercept. The line with no intercept implies infection at the time of birth, because it goes through 0% divergence at age 0. The better-fitting line with the intercept shows a steeper increase in divergence. Regression with intercept: equation for the line: estimated % divergence while excreting *Y* = 0.112 *x, x* = age (months), *Y* = estimated % divergence, *R*^2^ = 0.85. Regression without intercept: equation for the line: estimated % divergence while excreting *Y* = 0.192*x* – 5, where *x* = age (months), *Y* = estimated % divergence, *R*^2^ = 0.99.

The complete VP1 genomic region of serotype 3 poliovirus (PV3) isolated from the child under study showed highly diverged sequences. The number of nucleotide mutations has been expressed as the percentage of changes with respect to the serotype 3 Sabin virus (Sabin3) genome, which varied from 41 (4.5% divergence) in the first sample at 4 years of age to a maximum 93 (10.33% divergence) in the 10th sample at 5 years of age (Table 2). There was a lot of variability observed between the samples, with the 10th sample showing disproportionately high sequence divergence, which was markedly reduced in the 11th sample (Figure 1). No recombination was observed in the VP1 gene. No poliovirus could be detected from the 14th sample onwards to date.

**Table 2:** History of virus isolation from the SCID patient.

**Sample no**.	**Age in months**	**Date of collection**	ITD^*^ result	**Sequencing result**	**No. of nucleotide changes**	**% Divergence from sabin3**
1	50	04-09-2014	P3DIS^†^	P3VDPV	41	4.56
2	53	04-12-2014	P3DIS	P3VDPV	46	5.11
3	55	26-02-2015	P3DIS	P3VDPV	49	5.44
4	56	30-03-2015	P3DIS	P3VDPV	48	5.33
5	58	25-05-2015	P3DIS	P3VDPV	55	6.11
6	61	04-08-2015	P3DIS	P3VDPV	61	6.78
7	63	05-10-2015	P3DIS	P3VDPV	64	7.11
8	65	02-12-2015	P3DIS	P3VDPV	73	8.11
9	66	05-01-2016	P3DIS	P3VDPV	66	7.33
10	67	10-02-2016	P3DIS	P3VDPV	93	10.33
11	68	07-03-2016	P3DIS	P3VDPV	68	7.56
12	70	30-05-2016	P3DIS	P3VDPV	67	7.44
13	73	04-08-2016	P3DIS	P3VDPV	76	8.44
14	75	31-10-2016	Negative	Negative	Nil	Nil
15	76	21-11-2016	Negative	Negative	Nil	Nil^‡^

* *Intratypic differentiation*.

† *Poliovirus type 3 discordant*.

‡ *To date*.

### Analysis of T Cell Responses

At 16 months, the patient was diagnosed as SCID with T-B-NK+ immunophenotype. Although his T cell counts were below normal range up to the age of 4, 5 years onwards, the CD3+ T cell population continuously increased up to the age of 7 years (Table 3; Figure S1). During the poliovirus clearance period (60–77 months), he showed an increased total leucocyte count with significant increase in T lymphocyte population particularly in cytotoxic T lymphocytes as compared to the counts of other months. Interestingly, he had persistent inverse CD4:CD8 ratio, which decreased with the progress in age. The NK cell population was maintained at a higher level (~10-fold) throughout the study period. Overall, the child showed inconsistent lymphocyte pattern.

**Table 3:** Immune profile of the SCID patient from the time of diagnosis up to the age of 7 years^*^.

	**Result in time points (age in months)**								
	**16**	**29**	**Normal range** **(0–3 yrs)**	**44**	**60**^†^	**Normal range** **(3–6 yrs)**	**77**^‡^	**87**	**Normal range (6–12 yrs)**
ALC/mm^3^	3,753	6,647	3,600–890	3,400	3,355	2,300–5,400	9,255	6,360	1,900–3,700
**LYMPHOCYTE SUBPOPULATION [ALC/mm**^**3**^**]**									
CD19+/B lymphocytes	150	598	720–2,600	884	101	390–1,400	185	191	270–860
CD3+T lymphocytes	1,989	2,061	2,100–6,200	1,054	1,845	1,400–3,700	6,664	4,833	1,200–2,600
CD3+/CD4+ Th lymphocytes	826	465	1,300–3,400	816	336	700–2,200	1,111	700	650–1,500
CD3+/CD8+ Tc lymphocytes	826	997	620–2,000	204	1,007	490–1,300	3,610	2,798	370–1,100
CD3–/CD16+56+ NK Cells	1,576	3,789	180–920	1,360	1,275	130–720	2,314	1,272	100–480

* *Surface markers for flow cytometry contained FITC-labeled CD3, PE-labeled CD16, and CD56, PerCP. Cy^TM^5.5-labeled CD45, PE-Cy^TM^7-labeled CD4, APC-labeled CD19, and APC-Cy7–labeled CD8. The samples were collected twice and experiments were repeated to rule out the inconsistency in the lymphocyte pattern. To maximize instrument performance and minimize inter-assay variability, CST (Cytometer Set-up and Tracking) beads were run daily on BD Facs-Aria machine. Yrs, years; ALC, absolute lymphocyte count*.

† *Lymphocyte count of the last sample collected when the child was shedding VDPV3*.

‡ *Lymphocyte count of the first sample collected after the child stopped VDPV3 shedding*.

### Analysis of B Cell Responses and Ig Levels

No significant increase in CD 19+ B cells was observed throughout the study. The serum Ig levels of the patient remained below normal range for all four types (IgG, IgM, IgA, and IgE) of Igs tested, and no substantial increase was observed correlating with the low CD19+ B cell count (Table 4).

**Table 4:** Quantification of serum Ig concentration of the SCID patient at different time points.

	**Age in months**						
	**16**	**29**	**44**	**59**	**77**	**87**	**Normal range**
IgG, g/L	<1.41^*^	2.23	9.95^†^	0.12	0.5	0.99	3.5–16.2
IgA, g/L	<0.244	NA	0.47^†^	0.002	0.004	0.004	0.17–3.18
IgM, g/L	0.203	0.206	–	–	<0.168^*^	<0.168^*^	0.30–2.65
IgE, IU/ml^‡^	<15.3^*^	<15.3^*^	–	–	<4.45^*^	<17.8^*^	3–423

* *Serum Ig quantified by nephelometry (below cutoff range). Values without star mark were quantified by IgG/A ELISA (absolute values)*.

† *Sample collected after IVIG administration*.

‡ *International Units/milliliter*.

### Analysis of Cytokine/Chemokine Release

**Figure 2:**
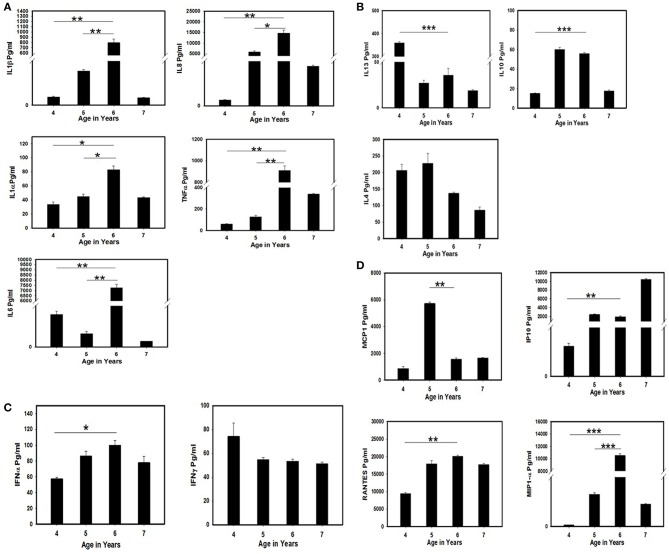
Estimation of cytokine/chemokine levels in the serum samples of the SCID patient tested positive for VDPV3 and abruptly stopped poliovirus shedding after 2 years of continuous excretion at the age of 6 years. The serum samples collected from the child during poliovirus excretion period (4 and 5th year) and after viral clearance (6 and 7th year) were tested by multiplex cytokine ELISA. The assays were performed twice and the mean and standard error were considered for evaluating the statistical significance by Student's *t-*test. ^*^ represents *p* < 0.05, ^**^ represent *p* < 0.01, and ^***^ indicate *p* < 0.001. **(A)** Estimation of pro-inflammatory cytokine levels. **(B)** Estimation of Th2 cytokine levels. **(C)** Estimation of interferon levels. **(D)** Estimation of chemokine levels in the SCID patient before and after poliovirus clearance.

The serum samples collected at 1-year intervals (approximately) for 4 years for the follow-up of the immunological parameters of the patient were tested for cytokine secretion in order to investigate the role of specific cytokines responsible for virus clearance. Significantly high release of pro-inflammatory cytokines such as IL-6, IL-8, IL-1β, IL-1α, and TNF (Figure 2A) was detected when compared between the 4/5th (iVDPV3 excretion period) and 6th year (iVDPV3 cleared) of the child. A drastic difference in MCP-1 and MIP-1α levels was also observed wherein a significant decrease and increase in cytokine levels were observed between the 5 and 6th year only, respectively (Figure 2D). IL13, IL10, IFNα, IP-10, and RANTES levels showed a significant difference at 4 years of age when compared to other years, but no significant difference could be observed between the 5 and 6th year during which the child stopped poliovirus shedding (Figures 2B,C).

## Discussion

### Molecular Characteristics of iVDPV Infection

The patient was excreting VDPV3 with 4.56% sequence drift from reference Sabin3 strain, 4 years (approximately) after birth. He was immunized with OPV at the time of birth when his immunodeficiency condition was unknown. We presume that the first OPV exposure could have been the infective OPV dose considering 1% nucleotide change per year (4.5% in 4 years) (1). However, it does not preclude the possibility of any dose received or rule out community exposure. Based on the accumulation of nucleotide changes in the VP1 coding region in the next 2-year period (4–6th), it could be predicted that the nucleotide changes followed an abnormal pattern, which varied from 4 to 10% within a span of 2 years as opposed to 1% as shown in previous studies (21, 22). There is considerable uncertainty about when this child actually became infected with the type 3 virus, and it is possible that the child became infected following community exposure to a circulating OPV3-related virus that had already diverged some (i.e., this iVDPV is not a result of direct vaccination of the child, but secondary exposure from another infected individual). Figure 1 conveys some of the variability in the samples over time.

Further studies on the phylogenetic analysis of poliovirus evolution are planned to reveal the mechanism of strikingly high VP1 divergence rate.

### Changes in Innate Immune Response and Poliovirus Clearance

Spontaneous clearance of iVDPV infection in CID (combined immune-deficiency) patients with defects in cellular and humoral immunity could be partly attributed to the innate immune cells (6, 23). Consistently low levels of immunoglobulins were noted in the patient throughout the study period despite normal B cell numbers noted at 44 months of age, ruling out the possibility of any role of antibodies in poliovirus clearance.

NK cells work to control viral infections by secreting IFNγ and TNFα. Although we observed consistently increased number of NK cells throughout the study, the child did not clear VDPV up to 6 years of age. Similar findings with increased number of NK cells in patients with T-B-NK+SCID and persistent poliovirus infection have already been reported (24). A 10-fold increase in TNFα was observed when the child cleared infection, but the IFNγ secretion was either unchanged or decreased with increase in age indicating that the NK cells were either not activated or inhibited by certain receptors, which subsequently supressed IFNγ synthesis (25).

Enteroviruses are associated with chronic inflammatory and autoimmune diseases in humans. In these conditions, the cytokine network is supposed to have an important role in inflammation and modulation of the (auto) immune response. It has been demonstrated that PV1 and EV71 induce production/release of pro-inflammatory cytokines IL-1, IL-6, TNF-α, IL-8, IP-10, and RANTES but not IFNα and γ by leucocytes and monocyte-derived macrophages, respectively (26, 27). In our study, all the major pro-inflammatory cytokines along with the chemokines were significantly increased, indicating the presence of some auto-inflammatory condition that may have caused the clearance of poliovirus infection. A significantly higher level of MIP-1α (macrophage inflammatory protein 1α) indicates a major role of macrophages in the production of these inflammatory cytokines.

Inflammatory diarrheal illness has been reported to clear iVDPV infections (6, 28). It is important to note that during the 73rd month, the child was admitted to the hospital for norovirus diarrhea for 1 month, after which he cleared iVDPV3 infection. Therefore, we presume that some previous or ongoing bacterial or viral pathogens recognized by certain TLRs/RLRs found in the body could have induced macrophages to produce inflammatory cytokines, which cleared infection by destructing the GI epithelial lining or infected enterocytes (7).

### Changes in Adaptive Immune Response and Viral Clearance

Several reports have shown that the PID patient with hypogammaglobulinemia resulted in persistent excretion of VDPV for years, indicating that viral clearance is mediated by antibodies (8, 29). The SCID patient in our study had very low level of serum immunoglobulins but received regular IVIG therapy. But as reported in earlier studies, attempting to stop poliovirus excretion by IVIG therapy did not result in infection clearance (9, 24).

Studies have reported that T cell deficiency does not result in persistent viral excretion (30, 31). However, clearance of poliovirus infection by both CD4+ve and CD8+ve cytotoxic T lymphocytes by secretion of IFNγ has been shown by some earlier studies (32, 33). The child was initially labeled as leaky SCID as there was significant T cell lymphopenia but subsequently he had normal-high T cell counts with persistent low naive T cells, low TRECs, abnormal T cell proliferation, and oligoclonal T cells (Supplementary Appendix). He had significantly high levels of cytotoxic T cells that continued thereafter when he cleared poliovirus infection, indicating that cytotoxic effects of CD8+ T cells could have played a major role in infection clearance. However, like NK cells, these cells were also not activated enough as we did not find any increase in secretion of IFNγ in the serum samples before or after poliovirus clearance.

Our study is limited to retrospective analysis of the data based on which we hypothesized the mechanism of iVDPV3 clearance. Some confirmatory studies could not be performed since the required samples are not available. To confirm the inconsistent lymphocyte pattern of the SCID patient that might appear as technical error, the samples were collected twice and the experiments were repeated with proper calibration/quality control.

Asymptomatic iVDPV excretion in patients with CIDs has been reported particularly after the implementation of poliovirus screening program in recent years (34, 35). The patient in our study never had paralysis and his iVDPV infection was detected only by screening. His leaky SCID disorder with better survival chances as compared to classical SCID patients enabled prolonged iVDPV excretion increasing the risk. Our data highlight the importance of proper categorization of SCID patients (as per IUIS criteria) who are enrolled in poliovirus screening programs to assess the risk of iVDPV excretion in the polio endgame. OPV is still being used in India as part of a polio eradication and endgame strategy. WHO suggests administration of multiple doses of bivalent OPV and at least one dose of IPV, for all countries using OPV. It also suggests that patients who are severely immunocompromised with known underlying conditions should avoid vaccination with OPV (36). The PID incidence rate is not known in India. Introducing pre-natal diagnosis and/or newborn screening facilities in India should help with identifying PID patients before the birth dose of OPV is given, minimizing the risk of VDPV/VAPP.

In this study, we investigated the immune responses and characterized the iVDPV3 isolates of a leaky SCID patient who cleared infection, and discussed the possible immune mechanisms responsible for the sudden poliovirus clearance without receiving a stem cell transplant. Our findings suggest that in the absence of B cells and sufficiently activated T/NK cells, macrophages may produce auto-inflammatory conditions by TLR/RLR ligands, expressed by previous/ongoing bacterial or viral infection to clear iVDPV infections as reported by others (24). It also suggests that in the absence of a newborn screening facility, all the patients with combined immunodeficiency need to be monitored for poliovirus excretion, and if found positive, the immune profile should be tested intermittently in order to manage the risk of iVDPV excretion in the polio eradication endgame strategy.

## Data Availability

All datasets generated for this study are included in the manuscript/Supplementary Files.

## Ethics Statement

The study was carried out in accordance with the recommendations of the Ethics Committee of ICMR-National Institute of Virology and Bai Jerbai Wadia Children's Hospital, with written informed consent from the parents of the participant. The informed consent included the authorization to publish information such as age, gender, medical history, family history, and the investigation reports as these data were required for analyzing the molecular evolution of the virus.

## Author Contributions

MCM planned the experiments, supervised the study, analyzed the data, and wrote the manuscript. MRM supervised part of the study, analyzed the data, and reviewed the manuscript. JA, SV, DS, UN, SR, MG, SS, and AD were involved in performing the laboratory investigations. MD and PT supervised the clinical care of the patients and provided clinical information. JD conceived the idea and reviewed the manuscript.

### Conflict of Interest Statement

The authors declare that the research was conducted in the absence of any commercial or financial relationships that could be construed as a potential conflict of interest.
