# Supplementary material for: Natural Clearance of Prolonged VDPV Infection in a Child With Primary Immunodeficiency Disorder
Source: Front Immunol. 2019 Jul 23;10:1567. doi: 10.3389/fimmu.2019.01567 (PMC6663979; doi:10.3389/fimmu.2019.01567)
Supplement: Supplementary file 1 [file Data_Sheet_1.docx]

**Supplementary Appendix**

**Table of Contents**

**Contents Page No.**

Methods 2

**Tables**  3

S1 (T naïve and memory cell workup)

S2 (HLA-DR activation study)

**Figure** 4

S1 (Lymphocyte subset trend)

**Methods:**

**T naïve and memory cell workup**

The percentage of naive T cell subsets on CD4 and CD8 cells was measured by flow cytometric evaluation of CD45RA, CD62L using anti-CD45RA phycoerythrin (PE) and anti- CD62L allophycocyanin (APC) purchased from BD Biosciences, San Jose, CA, USA by FACS Aria I flow cytometer and FACS Diva Software (BD Biosciences, San Jose, CA, USA).

**HLA-DR Expression study**

The patient was evaluated for cell surface markers specific for activated T cells (anti-CD3 fluorescein isothiocyanate; FITC), anti HLA-DR (anti-HLA DR Phycoerythrin;PE) purchased from BD Biosciences, San Jose, CA, USA by using FACS Aria I flow cytometer and FACS Diva Software (BD Biosciences, San Jose, CA, USA).

| **Table-S1: Percentage of Naïve T cell population in the SCID patient *** | | | |
| --- | --- | --- | --- |
| **Age in Months** | **Lymphocyte Subpopulation** | **% Th cells (Normal range , 50-85)** | **% Tc cells (Normal Range, 42-81)** |
| **64** | **Naïve T cells** | **26** | **0.4** |
| **79** | **Naïve T cells** | **1.5** | **0.7** |
| **96** | **Naïve T cells** | **0.3** | **0.4** |

***Majority of CD3+ T cells (99%) have CD45RO+ phenotype.**

| **Table S2: Percentage of activated T cells in the SCID patients** | | |
| --- | --- | --- |
| Sr. No | Lymphocyte  Subpopulation | Result (%) |
| 1 | CD3^+^/ HLA-DR^+^  (% T cells) | 74 |

**Figure S1**
